# Comparative Efficacy of Autologous Hematopoietic and Mesenchymal Stem Cell Transplantation in Patients with Systemic Sclerosis: A Systematic Review

**DOI:** 10.3390/jcm15010261

**Published:** 2025-12-29

**Authors:** Saltanat Bakirova, Abai Baigenzhin, Saltanat Tuganbekova, Manarbek Askarov, Elmira Chuvakova, Marlen Doskali, Ainur Doszhan

**Affiliations:** 1 Department of Internal Diseases #4, NCJSC “Astana Medical University”, Astana 010009, Kazakhstan; info@kidney.kz; 2 Department of Therapy #2, JSC «National Scientific Medical Center» Astana, Astana 010009, Kazakhstan; abai.baigenzhin@outlook.com (A.B.); askarov.manarbek@outlook.com (M.A.); elkatoma11@gmail.com (E.C.); ainurdoszhan8@gmail.com (A.D.); 3 Department of Cell Therapy, Tokyo Relife Clinic Innovative Cellular Research and Regenerative Therapy Center, Tokyo 104-0061, Japan; mdoskali@tokyo-relife.com

**Keywords:** systemic sclerosis, hematopoietic stem cell transplantation, mesenchymal stem cells, autoimmune disease, mRSS, pulmonary function, clinical outcomes

## Abstract

**Background/Objectives**: Systemic sclerosis (SSc) is a rare and severe autoimmune disease with limited treatment options. Autologous hematopoietic stem cell transplantation (HSCT) and mesenchymal stem cell transplantation (MSCT) have emerged as promising therapeutic strategies, especially for patients with refractory or rapidly progressive forms of the disease. However, no comparative synthesis has yet evaluated the clinical outcomes, safety, and applicability of these two distinct stem-cell-based interventions. This systematic review aimed to perform a comparative qualitative synthesis of clinical outcomes, safety profiles, and evidence quality for HSCT and MSCT in patients with systemic sclerosis, focusing on survival, skin fibrosis, pulmonary function, and adverse events. **Methods**: A comprehensive search was conducted in PubMed, ScienceDirect, Cochrane Library, and Google Scholar for the period between 2015 and May 2025. Studies were included if they reported on adult patients with a confirmed diagnosis of SSc treated with either autologous HSCT or MSCT and provided clinical outcome data. Risk of bias was assessed using the Newcastle-Ottawa Scale. Due to heterogeneity across studies, results were synthesized qualitatively. **Results**: Eleven studies met the inclusion criteria, comprising 504 patients (316 HSCT, 188 MSCT). HSCT showed consistent improvement in survival (1-, 5-, and 10-year), reduction in modified Rodnan skin scores (mRSS), and s ilization or improvement in pulmonary function (DLCO, FVC), albeit with a higher incidence of serious adverse events, including transplant-related mortality (up to 10%) and infectious complications. MSCT demonstrated favorable effects on skin fibrosis and lung involvement with a significantly lower toxicity profile. However, long-term survival data and methodological robustness were limited were more limited. HSCT was supported by multiple randomized controlled trials and international guidelines, while MSCT remains under clinical investigation with promising but still preliminary evidence. **Conclusions**: Both HSCT and MSCT demonstrate potential clinical benefits in systemic sclerosis, but they differ substantially in evidence strength and risk profiles. HSCT provides the most robust evidence for long-term disease modification in carefully selected patients, whereas MSCT represents a promising and safer investigational option, particularly for patients ineligible for intensive therapy. Further well-designed comparative studies are required to define their optimal clinical roles.

## 1. Introduction

Systemic sclerosis (SSc) is a chronic autoimmune rheumatic disease of unknown origin associated with environmental, hormonal and genetic factors, including epigenetic mechanisms and microRNAs [1,2,3]. The disease is characterized by damage to small vessel endothelial cells, increased vascular permeability, fibroblast activation and excessive extracellular matrix deposition [4,5]. This leads to widespread inflammation, visceral fibrosis, skin thickening and vasculopathy [6,7].

SSc is considered a rare disease, with an estimated incidence of 0.77 to 5.6 per 100,000 populations per year and a prevalence of approximately 23 per 100,000 (95% CI: 16–29) [8,9]. Mortality in this disease is significantly increased, with standardized mortality rates up to 2.72 times higher than in the general population [10].

Traditional immunosuppressive therapy (cyclophosphamide, methotrexate, mycophenolate mofetil) provides limited disease control and is often associated with significant side effects [11]. Targeted therapy, including monoclonal antibodies or tyrosine kinase inhibitors, is currently in early clinical trials, but no universal standard has been established [12,13]. Thus, the treatment of SSc remains an unsolved clinical problem.

An important step in therapy was the development of autologous hematopoietic stem cell transplantation (HSCT) in the late 1990s and early 2000s, aimed at restoring the immune system in patients with rapidly progressing, refractory disease [14,15,16]. Randomized controlled trials, including the ASSIST, ASTIS, and SCOT studies, have demonstrated the superiority of HSCT over conventional immunosuppressive therapy in selected high-risk patients, particularly those with diffuse cutaneous SSc (DCSSc) and early organ involvement [17,18,19]. However, HSCT carries significant treatment-related risks and is contraindicated in patients with advanced cardiac, pulmonary, or renal disease [20].

Recently, mesenchymal stromal cell (MSC) therapy has emerged as a potential alternative due to its immunomodulatory, anti-fibrotic, and pro-angiogenic properties [21,22,23]. MSCs can be derived from a variety of sources, including bone marrow, adipose tissue, and umbilical cord, and exert their therapeutic effects primarily through paracrine mechanisms [24]. Several early-phase studies and small clinical trials have demonstrated improvements in skin fibrosis, lung function, and quality of life after MSC [25,26,27]. Compared with HSCT, MSC is less invasive and may be more suitable for patients with contraindications to conditioning regimens [28].

Despite the growing interest in both HSCT and MSC, no head-to-head comparisons have been conducted. Furthermore, published studies are heterogeneous in design, patient selection, treatment protocols, and outcome measures [29]. To date, the relative efficacy and safety profile of these two regenerative strategies remain unclear [30]. Most existing reviews focus on one treatment modality in isolation, without systematically comparing it with other cell therapies [29,31,32,33,34].

The aim of this systematic review is to provide a comprehensive synthesis of the available evidence on hematopoietic stem cell transplantation (HSCT) and mesenchymal stromal cell therapy (MSCT) for the treatment of systemic sclerosis. Specifically, this review aims to perform a comparative qualitative synthesis of clinical outcomes, safety profiles, and strength of evidence for both treatment modalities, focusing on survival, skin fibrosis, pulmonary function, and adverse events. By systematically contrasting these outcome domains and methodological characteristics, this review seeks to clarify the current clinical role of HSCT and MSCT, identify key limitations of the existing evidence, and outline priorities for future research in systemic sclerosis.

## 2. Materials and Methods

The study protocol was registered in the PROSPERO International Prospective Register of Systematic Reviews of the National Institute for Health Research (ID: CRD420251114759) [35].

### 2.1. Search Strategy

An initial search of the PROSPERO database for registered protocols of comparable systematic reviews and meta-analyses identified no relevant registration. This systematic review was conducted and reported in accordance with the PRISMA 2020 (Preferred Reporting Items for Systematic Reviews and Meta-Analyses) guidelines [36]. The completed PRISMA checklist (Table S1) are provided in the Supplementary Materials.

A comprehensive search was performed to identify relevant studies published between January 2015 and 1 May 2025, in order to capture current advancements in cell-based therapies for systemic sclerosis. The following databases were systematically searched: PubMed, Cochrane Library, Science Direct, and Google Scholar. Google Scholar was included as a complementary source to capture potentially relevant grey literature and recently published studies not yet indexed in major databases. Reference lists of included full-text articles and relevant reviews were also manually screened to ensure completeness of the search. All records were imported into reference management software, and duplicates were identified and removed prior to screening.

The specific search terms used included combinations of the disease (e.g., “systemic sclerosis”, “scleroderma”) and therapeutic approaches (e.g., “autologous hematopoietic stem cell transplantation”, “mesenchymal stem cells”, “auto-HSC”, “auto-MSC”). Boolean operators (AND, OR) were applied to refine the queries. The selection of search strategies was adapted to the interface of each platform. The search strategies for each database are summarized in the Supplementary Materials Table S2.

**Figure 1:**
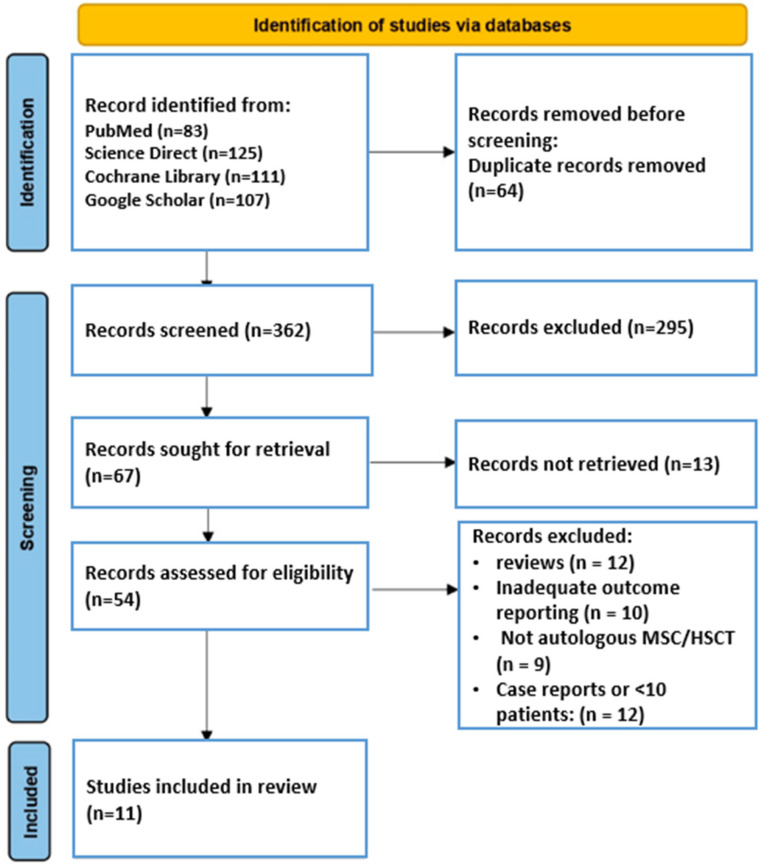
Flow chart of the PRISMA study selection process.

Total number of articles identified across all databases: 426. After removing duplicates and screening titles and abstracts, 54 full-text articles were assessed for eligibility. Based on the predefined inclusion and exclusion criteria (outlined in Section 2.2), only 11 studies met all inclusion criteria and were included in the final review (Figure 1).

### 2.2. Eligibility Criteria

This systematic review included studies based on predefined inclusion and exclusion criteria to ensure methodological consistency and relevance to the research objectives. The criteria were as follows: Studies were eligible for inclusion if they investigated either autologous hematopoietic stem cell transplantation (HSCT) or mesenchymal stromal cell therapy (MSCT) in patients with systemic sclerosis (SSc), provided that MSCT adhered to the minimum criteria established by the International Society for Cellular Therapy (ISCT). Eligible cell types included both CD34^+^-selected and unselected autologous hematopoietic stem cells, as well as cultured mesenchymal stromal cells derived from bone marrow, adipose tissue, or umbilical cord sources.

Patients had to have a confirmed diagnosis of SSc based on the ACR 1980 or ACR/EULAR 2013 classification criteria. Only adult patients (aged 18 years and older) of both sexes were included. Studies involving mixed autoimmune populations were only included if they provided a separate analysis of the SSc subgroup. The review focused on clinical studies—randomized controlled trials (RCTs), prospective or retrospective cohort studies, and open-label clinical trials. Case reports, studies with fewer than 10 participants, reviews, editorials, letters, and preclinical studies (in vitro or animal experiments) were excluded. A minimum follow-up period of 6 months’ post-transplantation or intervention was required to assess medium- to long-term clinical outcomes. Studies without clear clinical outcome reporting-such as modified Rodnan skin score (mRSS), pulmonary function tests (FVC, DLCO), survival, or patient-reported quality of life-were excluded. The complete eligibility framework is summarized in Table 1.

### 2.3. Selection of Studies and Data Extraction

After removal of duplicates, two independent reviewers conducted a stepwise screening of all retrieved records by evaluating titles and abstracts for relevance. Studies that met the inclusion criteria were then selected for full-text review. The final decision regarding inclusion was made after thorough examination of the full articles against the pre-defined eligibility criteria outlined in Section 2.2.

Data extraction was independently performed by two reviewers using a standardized data collection form. Any discrepancies in interpretation or data coding were resolved by consensus or, if needed, through discussion with a third senior reviewer. The following data were extracted from each eligible study: first author’s name, year of publication, country of origin, study design, type of stem cell therapy (autologous hematopoietic stem cell transplantation [HSCT] or mesenchymal stromal cell therapy [MSCT]), source and type of cells used, number and characteristics of patients (including mean age and SSc subtype), duration of follow-up, and reported clinical outcomes.

Clinical endpoints included skin involvement (mRSS), pulmonary function tests (FVC, DLCO), digital ulcers, Raynaud’s index, hand function (CHFS) and adverse events, including serious adverse events (SAEs). For MSCT studies, data on specific scoring systems such as MHISS and VAS were also collected where available. If any outcome data were missing or unclear, additional information was retrieved manually from the article text, tables, or Supplementary Files. All extracted data were reviewed for accuracy and consistency, and then compiled into structured summary tables for comparative and narrative analysis.

### 2.4. Risk of Bias (Quality) Assessment

The methodological quality of the included studies was evaluated using the Newcastle-Ottawa Scale (NOS), a validated tool for the assessment of non-randomized studies [37]. The NOS examines three domains: Selection of participants (maximum 4 points), Comparability of groups (maximum 2 points), and Outcome assessment (maximum 3 points), with a maximum score of 9. Higher scores indicate superior methodological quality and lower risk of bias. Two reviewers (S.T., M.A.) independently performed the assessments; disagreements were resolved by discussion and, if necessary, consultation with a third reviewer. Studies scoring ≥ 7 points were categorized as high quality, those scoring 5–6 as moderate quality, and those scoring ≤ 4 as low qualities. Detailed scoring for each study is provided in Supplementary Table S3.

We did not exclude studies based on NOS ratings, but results from those with lower quality scores were interpreted with caution. Overall, HSCT trials-particularly randomized controlled trials and large registry-based studies-consistently demonstrated high scores (8–9/9), indicating a low risk of bias, whereas most MSCT trials were retrospective, early-phase or small single-center studies, with lower scores (6–7/9), reflecting a moderate-to-high risk of bias.

Given the marked heterogeneity in study design (randomized versus observational), patient populations, disease severity, stem cell sources, conditioning regimens, follow-up duration, and outcome definitions, conducting a formal quantitative meta-analysis was not appropriate. As a result, traditional statistical approaches for evaluating publication bias, such as funnel plots or Egger’s regression, were not applicable. Nonetheless, the limited number of neutral or negative MSCT studies suggests a potential risk of selective reporting and underrepresentation of unfavorable results, which may have influenced the overall evidence profile.

To account for variability across studies, we pre-specified additional qualitative analyses. A subgroup assessment was undertaken to explore potential differences in treatment effects according to patient and disease characteristics. Furthermore, a sensitivity assessment was carried out by re-evaluating the overall conclusions after excluding smaller retrospective cohorts. These steps were intended to test the consistency and robustness of the findings in the presence of methodological heterogeneity.

## 3. Results

### 3.1. Included Study Characteristics

A total of eleven clinical studies met the inclusion criteria for this systematic review, comprising seven studies on hematopoietic stem cell transplantation (HSCT) [38,39,40,41,42,43,44] and four on mesenchymal stem cell transplantation (MSCT) [45,46,47,48]. Collectively, these studies included 504 patients diagnosed with systemic sclerosis (SSc), of whom 316 received HSCT and 188 received MSCT. All studies were published between 2015 and 2025 and met predefined quality thresholds in terms of study design, clarity of outcomes, and methodological rigor. Quality assessment using the Newcastle-Ottawa Scale indicated consistently high methodological quality among HSCT trials (8–9 points), particularly for randomized and registry-based studies, whereas MSCT studies generally scored lower (6–7 points), reflecting moderate-to-high risk of bias (Table S3).

The HSCT cohort was predominantly composed of patients with rapidly progressive diffuse cutaneous systemic sclerosis (dcSSc), most of whom were refractory to conventional immunosuppressive therapy [38,39,41,42]. In contrast, the MSCT cohort included patients with both moderate and severe forms of SSc, including those with early-stage disease or contraindications to high-intensity treatment regimens [45,46,47,48].

The duration of follow-up in HSCT studies ranged from 12 months to 15 years [38,39,40,41,42,43,44], enabling assessment of both early and long-term outcomes such as survival, disease progression, and treatment-related toxicity. MSCT studies, while more recent, offered substantial longitudinal data as well, with follow-up periods spanning 12 months to 10 years [45,46,47,48]. This allowed meaningful evaluation of sustained clinical benefits, organ-specific responses, and overall safety.

Patients in HSCT trials were generally younger, with median or mean ages ranging from 23 to 47 years [38,39,40,41,42,43,44], reflecting stringent eligibility criteria related to the risk of transplant-related complications. In MSCT studies, patient age ranged approximately from 18 to 47 years [45,46,47,48], indicating a broader age variability compared with HSCT cohorts and reflecting less restrictive inclusion criteria. Across all studies, the majority of patients were female, consistent with the known sex distribution of systemic sclerosis.

Stem cell sources and processing techniques varied between the two treatment modalities. In HSCT studies, peripheral blood CD34^+^ stem cells were commonly used, with several trials employing positive selection of CD34^+^ cells to reduce autoreactive lymphocytes [38,41,42,44]. MSCT studies utilized mesenchymal stem cells derived from allogeneic sources such as umbilical cord or bone marrow, administered without the need for myeloablative conditioning [45,46,47,48]. Notably, MSCT protocols were associated with a more favorable toxicity profile, offering a safer therapeutic alternative for patients with significant internal organ involvement or those ineligible for HSCT.

Overall, the included studies represent a clinically significant and methodologically diverse body of evidence evaluating the use of stem cell-based therapies in systemic sclerosis. Comprehensive study characteristics and individual outcomes are summarized in Table 2.

### 3.2. Clinical Outcomes and Comparison of Cellular Therapy

The analysis of clinical outcomes across the eleven included studies revealed consistent improvement in key parameters of systemic sclerosis (SSc) following both autologous hematopoietic stem cell transplantation (HSCT) and mesenchymal stem cell therapy (MSCT). However, the scope and magnitude of clinical benefit varied between modalities and patient populations (Table 3).

In the HSCT group, most studies focused on patients with rapidly progressive diffuse cutaneous SSc (dcSSc), refractory to conventional immunosuppressive therapy. Across studies [38,39,40,41,42,43,44], HSCT led to significant improvements in skin fibrosis, lung function, and survival. For instance, in the landmark ASTIS trial by Sullivan et al. [38], event-free survival (EFS) was significantly higher in the HSCT arm (79% vs. 50%), with a corresponding improvement in overall survival (OS: 86% vs. 51%) over 54–72 months. Similarly, the study by Henes et al. [39] demonstrated a 2-year progression-free survival of 81.8% and OS of 90% in a real-world multicenter setting. Other studies, such as those by Georges et al. [41] and Blank et al. [43], confirmed the long-term durability of the response, with 5- to 15-year survival exceeding 85%.

HSCT also yielded functional improvements. In the Brazilian study by Costa Pereira et al. [44], significant enhancement in range of motion, CHFS, DASH scores, and six-minute walk test was observed, along with a notable decrease in mRSS (mean reduction of 8.3 points, *p* < 0.01). However, treatment-related mortality (TRM) varied from 0% to 10% depending on conditioning regimens and patient comorbidity, particularly in those with cardiac or renal involvement [39,41,42].

MSCT studies involved more heterogeneous populations, including moderate-to-severe SSc patients and those ineligible for HSCT. Alip et al. [45] reported a significant decrease in mRSS over five years and stabilization of pulmonary arterial hypertension (PAH), with no recorded adverse events. Yuan et al. [46] demonstrated a 10-year OS benefit in the MSC group (89.4% vs. 73.4%, *p* = 0.002) within a propensity score–matched observational cohort, with the most pronounced effects seen in female patients and those with PAH and ILD. The combination therapy trial by Zhang et al. [47] showed a reduction in mRSS (from 20.1 to 13.8), improved CT features, and downregulation of profibrotic cytokines (TGF-β, VEGF), while the phase 1/2 study by Farge et al. [48] highlighted stabilization of lung function and a ≥25% mRSS reduction in 75% of patients.

Notably, the safety profile of MSCT was favorable across all studies [45,46,47,48], with no treatment-related deaths or severe immune complications reported. By contrast, HSCT was associated with serious adverse events in a subset of patients, particularly those with high-risk profiles, underscoring the importance of careful patient selection.

A comparative synthesis of the clinical data is presented in Table 4. Hematopoietic stem cell transplantation (HSCT) demonstrates consistently strong evidence across multiple outcomes, particularly in terms of long-term survival and disease modification. Six out of seven studies reported improved survival, with follow-up periods extending up to 15 years. Significant reductions in skin fibrosis (mRSS) and stabilization or improvement in pulmonary function (DLCO/FVC) were also consistently observed.

Secondary functional and patient-reported outcomes were reported only in a limited subset of studies and were assessed using heterogeneous instruments. Improvements in functional status and quality of life following HSCT were described by Sullivan et al. [38] and Costa Pereira et al. [44], including reductions in HAQ-DI, improvements in hand function (CHFS), range of motion, and patient-reported quality-of-life measures (SF-36, DASH). Among MSCT studies, Alip et al. [45] reported reductions in HAQ-DI, finger ulcer burden, and improved hand function, while Zhang et al. [47] described improvement in Raynaud’s index; however, these outcomes were inconsistently reported and were not primary endpoints in most studies.

In addition to the primary outcome synthesis, we performed a narrative subgroup assessment. Evidence across HSCT studies suggests that the greatest benefits are observed in younger patients with early diffuse cutaneous systemic sclerosis and preserved cardiopulmonary function [38,39,41,43]. Conversely, MSCT trials indicate potential effectiveness in women, younger individuals, and in patients with pulmonary arterial hypertension or interstitial lung disease [46]. Narrative sensitivity analysis demonstrated that excluding smaller retrospective cohorts (Dong, 2022 [40], Zhang, 2017 [47]) did not alter the overall direction of results, supporting the robustness of the main findings despite methodological heterogeneity.

To address the heterogeneity of outcome reporting across studies, we standardized the key clinical endpoints (mRSS, FVC, DLCO, survival, functional outcomes, and safety) and summarized them in Table 5. This comparative overview highlights both the variability in methodology and the consistency of trends: HSCT studies demonstrated stronger evidence for long-term survival [38,39,40,41,42,43,44], whereas MSCT studies reported more favorable safety and functional outcomes [45,46,47,48].

Mesenchymal stem cell therapy (MSCT), while supported by fewer and more heterogeneous studies, showed meaningful improvements in mRSS and pulmonary function in the majority of cases. Moreover, MSCT studies more frequently reported patient-centered outcomes, such as hand function scores and pain assessment scales, reflecting their utility in improving quality of life.

From a safety standpoint, MSCT had a considerably more favorable profile, with no reports of treatment-related mortality. In contrast, HSCT was associated with substantial early risk, including transplant-related mortality (TRM) of up to 10%, particularly in older patients or those with organ involvement.

Table 6 summarizes the methodological and structural differences between HSCT and MSCT trials. While HSCT benefits from established international guideline support and a robust base of randomized controlled trials and registries, MSCT is still emerging, with smaller-scale studies and less standardized protocols. Nonetheless, MSCT remains a promising therapeutic alternative, particularly for patient’s ineligible for HSCT due to comorbidities or disease severity.

**Figure 2:**
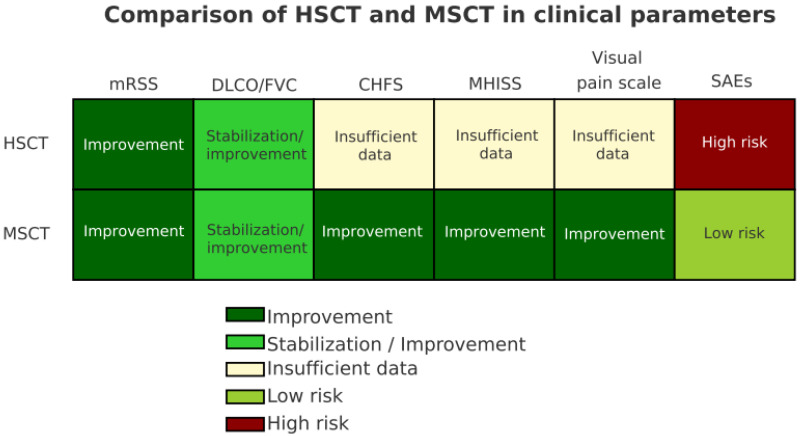
Comparative heatmap of HSCT and MSCT in systemic sclerosis. *The heatmap visually summarizes differences between HSCT and MSCT across survival benefit, skin fibrosis (mRSS), pulmonary function (FVC, DLCO), adverse events, and research quality. Darker shading represents stronger or more consistent evidence (e.g., ≥25% reduction in mRSS, ≥10% improvement in FVC or DLCO, or improved long-term survival confirmed across multiple studies). Lighter shading indicates inconsistent findings or insufficient evidence*.

To facilitate cross-study comparison, we generated a heatmap summarizing the relative strength of evidence for HSCT and MSCT across key clinical domains (Figure 2). Outcomes were graded according to standardized thresholds: ≥25% reduction in mRSS for skin fibrosis, ≥10% improvement in FVC or DLCO for pulmonary outcomes, and higher survival rates at 5–10 years. Adverse events and research quality were also scored. Darker shading indicates more consistent and clinically significant benefit across studies, whereas lighter shading reflects heterogeneity or weaker evidence).

## 4. Discussion

For patients with systemic sclerosis, particularly the diffuse cutaneous form, stem cell-based therapies provide promising alternatives to conventional immunosuppression. This review synthesized evidence from 11 clinical studies published between 2015 and 2025 [38,39,40,41,42,43,44,45,46,47,48], including 7 on hematopoietic stem cell transplantation (HSCT) and 4 on mesenchymal stem cell therapy, encompassing 504 patients.

HSCT studies [38,39,40,41,42,43,44] consistently demonstrated improvements in overall survival (OS), event-free survival (EFS), and skin fibrosis reduction, particularly in younger patients with severe, progressive disease. Randomized multicenter trials such as ASSIST [38] and ASTIS [39] provide the most robust data, further supported by registry-based studies [42,43]. However, HSCT carries substantial risks, including transplant-related mortality (TRM), infectious complications, and cardiovascular events [41,44].

Studies evaluating MSCT [45,46,47,48] reported favorable safety profiles, with no treatment-related mortality and generally reported improvements in skin involvement, pulmonary function (FVC, DLCO), and selected quality-of-life measures. In addition, longer follow-up in two observational cohorts [45,46] suggested disease stabilization and a potential association with reduced mortality, particularly among patients with pulmonary arterial hypertension or interstitial lung disease. However, these findings should be interpreted with caution, as MSCT studies were predominantly small, single-center, and non-randomized, resulting in lower methodological rigor and increased susceptibility to bias when compared with HSCT trials.

This difference in evidence quality was reflected in the formal risk-of-bias assessment. HSCT studies—especially randomized trials and national registry analyses-achieved high Newcastle-Ottawa Scale (NOS) scores (8–9/9), whereas most MSCT studies scored 6–7/9, indicating moderate-to-high risk of bias. Narrative subgroup synthesis suggested that HSCT appears to yield the greatest benefit in younger patients with early diffuse disease and preserved cardiopulmonary function, whereas MSCT may be associated with clinical improvement in selected subgroups, including women, younger patients, and those with cardiopulmonary involvement. Importantly, narrative sensitivity analyses demonstrated that exclusion of smaller retrospective cohorts did not materially change the overall direction of findings, supporting the internal consistency of the qualitative synthesis despite substantial heterogeneity. It must be emphasized that no head-to-head randomized trials directly comparing HSCT and MSCT have been conducted to date. Marked heterogeneity in study design, patient populations, baseline disease severity, outcome definitions, and duration of follow-up precluded formal meta-analytic pooling and limits direct quantitative comparison. Furthermore, the limited number of neutral or negative MSCT reports raises the possibility of publication bias, which may contribute to overestimation of treatment effects. These issues highlight the urgent need for standardized outcome definitions and harmonized reporting frameworks to enhance comparability across future studies.

Beyond whole-cell therapies, emerging research has explored alternative or adjunctive approaches, including mesenchymal stem cell-derived extracellular vesicles and exosomes [49,50,51], which may preserve immunomodulatory and antifibrotic properties while potentially reducing treatment-related risks [52,53,54,55]. In addition, combined or sequential strategies integrating cell-based therapies with immunosuppression have been proposed as potential means to improve durability of response and mitigate relapse [56,57,58,59], although clinical evidence in systemic sclerosis remains preliminary.

Taken together, the available evidence supports HSCT as the most evidence-based stem cell therapy for carefully selected younger patients with high disease activity and preserved organ function, while MSCT appears to represent a safer, investigational option for broader or more fragile patient populations [60,61,62]. Future randomized trials, prospective comparative studies, and international registries are essential to clarify relative efficacy, refine patient selection, and translate experimental promise into reliable clinical benefit.

### Limitations

This review has several limitations. First, substantial heterogeneity in study design, patient characteristics, stem cell sources, conditioning regimens, and outcome definitions precluded a formal meta-analysis and limited the ability to generate pooled effect estimates. Second, although most included studies met minimum quality thresholds, many-particularly those evaluating MSCT-were non-randomized, retrospective, and limited by small sample sizes, increasing the risk of bias and residual confounding. Third, key outcomes such as mRSS, DLCO, FVC, and safety parameters were inconsistently reported, and long-term follow-up was available only in selected cohorts, potentially underestimating late adverse effects or disease relapse. Finally, publication bias cannot be excluded, as negative or neutral studies may be underrepresented, and non-English or unpublished data might have been missed. These limitations emphasize the need for larger, high-quality, and standardized clinical trials to strengthen the evidence base for both HSCT and MSCT in systemic sclerosis.

## 5. Conclusions

Stem cell-based therapies represent an important and evolving treatment strategy for systemic sclerosis. This systematic review demonstrates that autologous hematopoietic stem cell transplantation is supported by high-quality randomized trials and long-term registry data, showing consistent benefits in survival and disease modification in selected patients with severe diffuse cutaneous disease. However, these benefits are counterbalanced by a substantial risk of treatment-related toxicity, underscoring the importance of stringent patient selection and specialized center expertise.

Mesenchymal stem cell transplantation, while supported primarily by non-randomized and observational studies, appears to be associated with improvements in skin fibrosis, pulmonary involvement, and patient-reported outcomes with a favorable safety profile. Current evidence for MSCT should be interpreted as preliminary and hypothesis-generating, as limitations in study design, sample size, and outcome standardization preclude definitive conclusions regarding long-term efficacy or survival benefit.

In the absence of direct comparative trials, HSCT should be considered the most evidence-based option for patients with rapidly progressive disease and preserved organ function, whereas MSCT may represent a safer investigational alternative for patients with contraindications to intensive immunoablation. Future research should prioritize head-to-head comparative studies, standardized outcome reporting, and long-term safety surveillance to refine patient selection and optimize therapeutic strategies.

## Figures and Tables

**Table 1:** Inclusion and exclusion criteria for publication selection.

Category	Inclusion Criteria	Exclusion Criteria
Type of intervention	− Autologous hematopoietic stem cell transplantation (HSCT) for SSc-Mesenchymal cell therapy (MSCT) that meets ISCT criteria	− Use of stromal vascular fraction (SVF)-Use of other cellular products (NK cells)
Cell type	− CD34^+^ selected or unselected autologous hematopoietic stem cells-Cultured MSCs from bone marrow, umbilical cord, adipose tissue, etc.	− Studies without specifying cell type or using unclassified cell populations
Diagnosis	− Confirmed diagnosis of systemic sclerosis (SSc) based on ACR 1980 or ACR/EULAR 2013 criteria	− Lack of reliable diagnosis of SSD-Mixed populations without separate analysis of patients with SSD
Type of research	− RCT (randomized controlled trials)-Prospective and retrospective cohort studies-Open clinical trials	− Case reports and studies with patients < 10, reviews, letters to the editor- Preclinical studies (in vitro/animal models),
Duration of observation	− Minimum 6 months after transplant/intervention	− Studies with follow-up less than 6 months
Outcomes	− Clinical outcomes (mRSS, FVC, DLCO, survival, quality of life, etc.) are indicated.	− No description of clinical outcomes
Publication period	− Publications for the period from 2015 to 2025	− Articles published before 2015
Publication language	− English	− Articles in other languages without translation
Full text	− Access to the full-text publication	− No access to the full text of the article

**Table 2:** General Characteristics of Included Studies.

No.	Author (Year)	Country	Type of Research	Cell Type	Source of Cells	*n* Patients/Avg Age	Form of SSD/Observation Period	Key Results
1	Sullivan et al. (2018) [38]	USA	RCT, multicenter.	HSC	CD34^+^ from the periphery	75 (36 + 39)/ ~45 years	Diffuse with ILD/54–72 months	▸ GRCS: 66.6% comparisons favor HSCT (*p* = 0.01); ▸ EFS 79% vs. 50% (*p* = 0.02); ▸ OS 86% vs. 51% (*p* = 0.02); ▸ ↓ mRSS and HAQ-DI
2	Hennes et al. (2021) [39]	Europe + Brazil	Prospective, multicenter	HSC	CD34^+^ (at 35/80)	80/ not specified	Severe dcSSc/ median 24 months	▸ 2 years PFS 81.8%; OS ~90%; ▸ response 88.7%; ▸ progression 11.9%; ▸ ↓ mRSS and ↑ FVC (*p* < 0.001)
3	Dong (2022) [40]	China	Retrospective	HSC	PBSC	12/ not specified	dcSSc/ median 12 months	▸ All achieved remission; ▸ 1 year OS 100%, PFS 90%; ▸ improvement of skin and lung symptoms
4	Georges et al. (2025) [41]	USA	Prospective, phase 2	HSC	PBSC (unmodified)	20/ median 34 years	dcSSc/ median 90 months	▸ 5 years OS ~85%; ▸ EFS 75%; DEFS 55%; ▸ patients without IBS and with SCF ≥75 mL/min showed sustained benefit
5	Strunz et al. (2021) [42]	Germany	Retrospective	HSC	CD34^+^-selected	22/ avg. 47.6 years	dcSSc/ median ~48 months	▸ SVR in 41%; ▸ associated with ↑ age and cardiac involvement; ▸ all SVR resolved without sequelae
6	Blank et al. (2022) [43]	Germany	National Register	HSC	CD34^+^-selected	80/ not specified	86.3% dcSSc/ up to 180 months	▸ 5/10/15 years OS: 96%/92%/86%; ▸ higher than controls (*p* = 0.041)
7	Costa Pereira et al. (2020) [44]	Brazil	Longitudinal cohort	HSC	PBSC	27/ 23.6 years	dcSSc/ 12 months	▸ Significant ↓ mRSS; ▸ improved ROM, hand strength, CHFS, DASH, mouth opening, 6MWT, SF-36
8	Alip et al. (2024) [45]	China	Retrospective	MSC	Umbilical cord blood	41/ 18.7 years	Moderate and severe SSD/ 60 months	▸ Significant ↓ mRSS at 1, 3, 5 years; ▸ PAH stabilized in 5/8; ▸ improved CT signs of ILD; ▸ ↓ HAQ-DI
9	Yuan et al. (2025) [46]	China	Cohort with PSM	MSC	Not specified	113 (MSC), 220 (control)/ median 47 years	dcSSc/ 120 months	▸ 10 years OS 89.4% vs. 73.4% (*p* = 0.002); ▸ HR 0.38 (95% CI 0.19–0.75); ▸ efficacy stronger in women, ≤47 years, with dcSSc, PAH, ILD
10	Zhang et al. (2017) [47]	China	Interventional	MSC + PF	Allogeneic	14/ avg. 37.4 years	dcSSc/ avg. 15.6 months	▸ ↓ mRSS at 12 mo; ▸ improved ILD and symptoms; ▸ ↓ autoantibodies, TGF-β, VEGF
11	Farge et al. (2022) [48]	France	Phase 1/2	MSC	Donor bone marrow	20/ 25 years	dcSSc/ 12 months	▸ 75% achieved clinical response (↓ mRSS ≥25% or ↑ FVC ≥10%); ▸ lung and cardiac function stabilized

*Abbreviations: HSCT—hematopoietic stem cell transplantation; HSC—hematopoietic stem cell; MSC—mesenchymal stem cell; PBSC—peripheral blood stem cell; CD34^+^—cluster of differentiation 34 positive cells; AHSCT—autologous hematopoietic stem cell transplantation; UMSCT—umbilical cord mesenchymal stem cell transplantation; SSD—systemic scleroderma; dcSSc—diffuse cutaneous systemic sclerosis; ILD—interstitial lung disease; PAH—pulmonary arterial hypertension; OS—overall survival; PFS—progression-free survival; EFS—event-free survival; DEFS—disease event-free survival; GRCS—global rank composite score; HAQ-DI—Health Assessment Questionnaire Disability Index; mRSS—modified Rodnan Skin Score; FVC—forced vital capacity; ROM—range of motion; CHFS—Cochin Hand Function Scale; DASH—Disabilities of the Arm, Shoulder and Hand questionnaire; 6MWT—six-minute walk test; SF-36—Short Form Health Survey; SVR—secondary autoimmune response; IBS—inflammatory bowel symptoms; SCF—stem cell factor; HR—hazard ratio; PSM—propensity score matching; PF—plasmapheresis; TGF-β—transforming growth factor beta; VEGF—vascular endothelial growth factor. The up and down arrows in this table mean increase and decrease.*

**Table 3:** Summary of comparative clinical outcomes between hematopoietic stem cell transplantation and mesenchymal stem cell therapy.

No.	Author (Year)	mRSS (Baseline → Follow-Up)	DLCO (%Pred)	FVC (%Pred)	Safety (Key Events)	Survival/EFS
1	Sullivan et al. (2018) [38]	29.7 ± 9.7 → ↓ (GRCS composite)	53.3 ± 7.9 → composite	74.1 ± 15.9 → composite	TRM 3% (54 mo), 6% (72 mo); infections, cytopenia	OS 86% vs. 51% (*p* = 0.02); EFS 79% vs. 50% (*p* = 0.02)
2	Henes et al. (2021) [39]	>15 (80%) → ↓ (*p* < 0.001)	>50% → ↑ (*p* < 0.001)	>70% → ↑ (*p* < 0.001)	TRM 6.25% (100 days); cardiotoxicity, CMV	2-year PFS 81.8%; OS ~90%
3	Dong et al. (2022) [40]	2–3 → 0–1	NR → improved	Restriction in 3 pts → improved	Mucositis, cytopenia; TRM 0	1-year OS 100%; PFS 90%
4	Georges et al. (2025) [41]	Median 34 → ≥50% ↓ (67%)	62 → ↑ ≥15% (39%)	76 → ↑ ≥10% (44%)	TRM 10%; SAEs ≥ G3 65%; ICU admission	5-year OS ~85%; EFS 75%; DEFS 55%
5	Strunz et al. (2021) [42]	Median 24 → ↓ 60–90%	NR	NR	Engraftment syndrome 41%; secondary autoimmunity 27%	OS not primary endpoint
6	Blank et al. (2022) [43]	17.6 ± 11.5 → 11.0 ± 8.5	53.9 → stable	NR	TRM 6.3%; SAEs variably reported	5/10/15-year OS: 96%/92%/86%
7	Costa Pereira et al. (2020) [44]	23.6 ± 12.3 → −8.3 (12 mo)	83.0 → ↓ ~7.7%	72.9 → stable	Febrile neutropenia 59%; CMV 29%; 1 fatal case	Survival not primary endpoint
8	Alip et al. (2024) [45]	18.7 ± 7.3 → 12.4 ± 8.5 (5 yrs)	NR → stable/improved (72% ILD)	NR	No TRM; no severe AEs	Not primary endpoint
9	Yuan et al. (2025) [46]	Median 13 → ↓ (subgroups)	NR	NR	NR	10-year OS 89.4% vs. 73.4% (*p* = 0.002); HR 0.38
10	Zhang et al. (2017) [47]	20.1 ± 3.1 → 13.8 ± 10.2	NR → improved (3 pts)	NR → improved (3 pts)	URTI (n = 5); no severe AEs	No deaths reported
11	Farge et al. (2022) [48]	24.7/26.0 → ↓ (both groups)	55.2/63.7 → stable	78.7/72.8 → stable	36 SAEs; none treatment-related	Clinical response 75%; OS not powered

*Abbreviations: mRSS—modified Rodnan Skin Score; DLCO—diffusing capacity of the lung for carbon monoxide; FVC—forced vital capacity; CHFS—Cochin Hand Function Scale; HAQ-DI—Health Assessment Questionnaire Disability Index; MHISS—Mouth Handicap in Systemic Sclerosis scale; VAS—Visual Analogue Scale; FTP—finger-to-palm distance; DASH—Disabilities of the Arm, Shoulder and Hand questionnaire; SF-36—Short Form Health Survey; N/V—nausea and vomiting; AE—adverse event; SAE—serious adverse event; TRM—treatment-related mortality; OS—overall survival; PFS—progression-free survival; EFS—event-free survival; DEFS—disease event-free survival; ILD—interstitial lung disease; GVHD—graft-versus-host disease; CMV—cytomegalovirus; URTI—upper respiratory tract infection. The up and down arrows in this table mean increase and decrease.*

**Table 4:** Comparative summary of clinical outcomes and research characteristics for HSCT vs. MSCT in systemic sclerosis.

Criterion	HSCT	MSCT	Commentary
Survival (1–5–10 years)	1 year OS 90–100% [38,39,40]; 5 years OS 79–96% [38,39,41,43]; 10 years OS 86–92% [43]	1 year OS 90–100% [45,46,47]; 5 years OS 73–89% [45,46]; 10 years OS 89% [46]	HSCT has more robust survival data, supported by RCTs and national registries; MSCT evidence mostly observational
Skin fibrosis (mRSS)	Significant ↓ in 6/7 studies [38,39,40,41,42,43,44]	Significant ↓ in all 4 studies [45,46,47,48]	Both therapies reduce skin thickening; HSCT shows larger effect sizes but with higher toxicity
Pulmonary function (FVC/DLCO)	Improvement or stabilization in 5/7 studies [38,39,40,41,42,43,44]	Improvement or stabilization in 3/4 studies [45,46,47]	HSCT and MSCT both show positive effects on ILD; reporting methods vary (absolute vs. % change)
Functional scores (CHFS/HAQ-DI/MHISS/VAS/SF-36)	Rarely reported (1 study, [44])	Reported in 2 studies [45,47]	MSCT studies tend to capture more patient-reported outcomes, HSCT literature underreports QoL measures
Adverse events (SAEs/TRM)	High risk: TRM up to 10% [38,39,40,41,42,43]; infections, cytopenia, cardiotoxicity, amenorrhea	Generally mild: no TRM [45,46,47,48]; mostly infusion reactions or transient symptoms	Safety profile clearly favors MSCT; HSCT requires strict patient selection and monitoring

*The downward arrow in this table means a decrease.*

**Table 5:** Standardization of clinical outcomes and methodological heterogeneity across included studies.

Outcome Measure	HSCT-Reported Range	MSCT-Reported Range	Assessment Methods Used	Notes on Heterogeneity
mRSS (skin fibrosis)	Reduction by 8–25 points over 1–5 years [38,39,40,41,42,43,44]	Reduction by 5–12 points over 1–5 years [45,46,47,48]	Modified Rodnan Skin Score (manual palpation, varied number of evaluators)	Significant variability in evaluator training and time-points; cut-offs for “clinically relevant improvement” inconsistent
FVC (% predicted)	Stabilization or improvement by +5–15% [38,39,40,41,42,43,44]	Stabilization or improvement by +3–10% [45,46,47,48]	Spirometry (techniques and calibration not always standardized)	Variation in reporting (absolute % vs. relative change)
DLCO (% predicted)	+5–20% improvement or stable values [38,39,40,41,42,43,44]	+3–12% improvement or stable values [45,46,47,48]	Single-breath DLCO	Lack of adjustment for hemoglobin
Overall survival (OS)	1-year: 90–100% [38,39,40,41,42,43,44]; 5-year: 79–96% [38,39,41,43]; 10-year: 86–92% [43]	1-year: 90–100% [45,46,47,48]; 5-year: 73–89% [45,46]; 10-year: up to 89% [46]	Kaplan–Meier analysis, registry follow-up	HSCT supported by RCTs and registries; MSCT mostly retrospective or single-center
Event-free survival (EFS/PFS)	2–5 years: 75–85% [38,39,40,41]	Rarely reported [45,46,47,48]	RCTs, registries	Absence of standardized definition of “event-free survival” across MSCT studies
Functional outcomes (CHFS, HAQ-DI, DASH, Raynaud’s index)	Reported in 1–2 studies only [44]	Reported more frequently (hand function, HAQ-DI, Raynaud’s score) [45,46,47,48]	Different scales and patient-reported outcomes	Limited comparability due to inconsistent endpoints
Adverse events	TRM up to 10% [38,39,40,41,42,43,44]; infections, amenorrhea, cardiotoxicity [38,39,40,41,42]	Mostly mild; no TRM; occasional transient adverse events [45,46,47,48]	WHO/CTCAE grading inconsistently applied	Strong contrast in safety reporting rigor (more detailed for HSCT, less standardized for MSCT)

**Table 6:** Comparison of Research Characteristics between HSCT and MSCT Trials.

Characteristic	HSCT	MSCT
Endorsement by international guidelines	Recommended by EULAR (2017, 2023) and ASBMT [38,39,40,41,42,43]	Not yet endorsed; current evidence insufficient [45,46,47,48]
Number of RCTs and systematic reviews	3 major RCTs [38,41] + ≥5 systematic reviews/meta-analyses	No RCTs; only cohort or phase I/II trials [45,46,47,48]
Follow-up duration	Long-term: up to 15 years in registry data [43]	Long-term: up to 10 years in one cohort [46]
Study scale	Multicenter prospective trials and national registries [38,39,40,41,42,43]	Mostly single-center, small-scale cohorts [45,46,47,48]

## Data Availability

The original contributions presented in this study are included in the article/Supplementary Material. Further inquiries can be directed to the corresponding author.

## Supplementary Materials

The following supporting information can be downloaded at: https://www.mdpi.com/article/10.3390/jcm15010261/s1, Table S1: The PRISMA checklist; Table S2: Search strategy; Table S3: Quality Assessment of Included Studies Using the Newcastle–Ottawa Scale (NOS).
